# The opposite end of the attention deficit hyperactivity disorder continuum: genetic and environmental aetiologies of extremely low ADHD traits

**DOI:** 10.1111/jcpp.12475

**Published:** 2015-10-17

**Authors:** Corina U. Greven, Andrew Merwood, Jolanda M. J. van der Meer, Claire M. A. Haworth, Nanda Rommelse, Jan K. Buitelaar

**Affiliations:** ^1^ Department of Cognitive NeuroscienceDonders Institute for BrainCognition and BehaviourRadboud University Medical CenterNijmegenThe Netherlands; ^2^ Karakter Child and Adolescent Psychiatry University CenterNijmegenThe Netherlands; ^3^ Medical Research Council Social, Genetic & Developmental Psychiatry CentreInstitute of Psychiatry, Psychology & NeuroscienceKing's College LondonLondonUK; ^4^ Department of PsychologyUniversity of BathBathUK; ^5^ School of Experimental Psychology and School of Social and Community MedicineMRC Integrative Epidemiology Unit at the University of BristolCoventryUK; ^6^ Department of PsychiatryDonders Institute for BrainCognition and BehaviourRadboud University Medical CenterNijmegenThe Netherlands

**Keywords:** Attention deficit hyperactivity disorder, quantitative trait, twin, extremes, positive genetics

## Abstract

**Background:**

Although attention deficit hyperactivity disorder (ADHD) is thought to reflect a continuously distributed quantitative trait, it is assessed through binary diagnosis or skewed measures biased towards its high, symptomatic extreme. A growing trend is to study the positive tail of normally distributed traits, a promising avenue, for example, to study high intelligence to increase power for gene‐hunting for intelligence. However, the emergence of such a ‘positive genetics’ model has been tempered for ADHD due to poor phenotypic resolution at the low extreme. Overcoming this methodological limitation, we conduct the first study to assess the aetiologies of low extreme ADHD traits.

**Methods:**

In a population‐representative sample of 2,143 twins, the Strength and Weaknesses of ADHD Symptoms and Normal behaviour (SWAN) questionnaire was used to assess ADHD traits on a continuum from low to high. Aetiological influences on extreme ADHD traits were estimated using DeFries–Fulker extremes analysis. ADHD traits were related to behavioural, cognitive and home environmental outcomes using regression.

**Results:**

Low extreme ADHD traits were significantly influenced by shared environmental factors (23–35%) but were not significantly heritable. In contrast, high‐extreme ADHD traits showed significant heritability (39–51%) but no shared environmental influences. Compared to individuals with high extreme or with average levels of ADHD traits, individuals with low extreme ADHD traits showed fewer internalizing and externalizing behaviour problems, better cognitive performance and more positive behaviours and positive home environmental outcomes.

**Conclusions:**

Shared environmental influences on low extreme ADHD traits may reflect passive gene‐environment correlation, which arises because parents provide environments as well as passing on genes. Studying the low extreme opens new avenues to study mechanisms underlying previously neglected positive behaviours. This is different from the current deficit‐based model of intervention, but congruent with a population‐level approach to improving youth wellbeing.

## Introduction

### Attention deficit hyperactivity disorder as a quantitative trait

Attention deficit hyperactivity disorder (ADHD) is a common neurodevelopmental disorder affecting around 6–7% of children and adolescents (Willcutt, 2012). Multifactorial disorders like ADHD are assumed to be influenced by many genes and environments of small effect resulting in a bell‐shaped quantitative trait distribution (Coghill & Sonuga‐Barke, 2012; Willcutt, 2005). Nonetheless, violating the assumption of a normal distribution, mental health disorders are assessed through binary diagnosis or measures biased towards the high, symptomatic extreme. Case–control studies typically lump together unaffected individuals in a single ‘no symptoms’ group, ignoring meaningful variation in the lower range of scores (Fair, Bathula, Nikolas, & Nigg, 2012). Further, traditional interview and questionnaire measures allow fine‐scaled assessment of variability at the high extreme of mental health continua, but attenuate variability at the low, unaffected end, reflected in skewed measure distributions. Overcoming this methodological limitation, this is the first study to assess the genetic and environmental aetiologies of low extreme ADHD scores.

### Towards a ‘positive genetics’ model of ADHD

An increasing trend in the literature is to focus on ‘positive genetics’; that is the study of adaptive tails of normally distributed traits, a promising avenue applied in the field of high intelligence to increase power for gene‐hunting for intelligence (Plomin & Deary, 2015). This research is based on the continuity hypothesis, that is, the strong evidence that distribution extremes of quantitative traits are influenced by the same genetic and environmental aetiologies affecting the rest of normal variation (Plomin, Haworth, & Davis, 2009). In contrast, the discontinuity hypothesis assumes that the aetiologies of distribution extremes differ qualitatively from the normal distribution; for example, severe cognitive disability and extreme height deviations are often caused by rare genetic abnormalities, whereas normal population variation in these attributes is thought to be influenced by many genes of small effect (Plomin & Deary, 2015; Shiang et al., 1994). Quantitative genetic DeFries‐Fulker (DF) extremes analysis (DeFries & Fulker, 1988) investigates genetic factors explaining why individuals at the distribution extreme, as a group, differ from the rest of the population, known as group heritability (h^2^g). Significant group heritability (h^2^g) that is similar in size to the heritability across the range of individual differences, suggests that extreme scores are heritable, and that there are genetic links between extreme scores and normal population variance, consistent with continuity. In contrast, nonsignificant h^2^g and significant heritability of individual differences would suggest that extreme scores are not significantly influenced by genetic factors, or that there are no genetic links between extreme scores and normal population variance, consistent with discontinuity. Like all common mental health continua (Plomin, DeFries, Knopik, & Neiderhiser, 2013), ADHD shows significant h^2^g (Larsson, Anckarsater, Råstam, Chang, & Lichtenstein, 2012; Willcutt, 2005), similar to heritability of individual differences in ADHD traits across the entire range of scores (around 70% for parent and teacher, somewhat lower for self‐ratings) (Greven, Asherson, Rijsdijk, & Plomin, 2011; Greven, Rijsdijk, & Plomin, 2011; Merwood et al., 2013; Willcutt, 2005). However, such an approach has still to be applied to low extreme ADHD traits.

### The low extreme: adaptive or maladaptive?

Whether low extremes of mental health continua represent positive (adaptive, desired) outcomes, or may also come with disadvantages remains a topic for empirical investigation (Plomin, 2013; Plomin et al., 2009). On the one hand, low extreme ADHD traits may be thought to reflect excellent attention, motor and impulse control coupled with good academic performance and low levels of comorbid behaviour problems. On the other hand, extreme deviations in either direction (low and high) on mental health continua may both represent maladaptive outcomes. Low extreme hyperactivity may reflect inertia or even behavioural rigidity; low extreme inattentiveness may reflect overfocusing and less flexible attentional shifting. From an evolutionary perspective, scoring in the midrange of the continuum may represent a favourable trade‐off between advantages and disadvantages of extreme polygenic liabilities (Nettle, 2006). Evidence suggests that personality dimensions related to ADHD (Parker, Majeski, & Collin, 2004) that are traditionally regarded as adaptive (e.g. extraversion, emotional stability, agreeableness, conscientiousness) may become dysfunctional when one has ‘too much of a good thing’ (McCord, Joseph, & Grijalva, 2014). For example, for introversion/extraversion personality traits, extreme scores in either direction have been shown to be associated with worse performance outcomes (Grant, 2013). Very high levels of conscientiousness share considerable overlap with obsessive‐compulsive tendencies, and are related to more negative effect, lower well‐being and more adverse reactions to negative life events (Carter, Guan, Maples, Williamson, & Miller, 2015). However, such a trade‐off hypothesis remains to be tested for ADHD traits.

### The current study

Few measures exist that provide resolution at the low extreme of mental health continua. For ADHD, the Strength and Weaknesses of ADHD Symptoms and Normal behaviour (SWAN) questionnaire (Hay, Bennett, Levy, Sergeant, & Swanson, 2007) assesses ADHD traits on a continuum from low to high (Arnett et al., 2011; Hay et al., 2007; Van der Meer et al., 2014). Here, we obtained self‐reports on the SWAN from a population sample of 2143 adolescent twins with two aims: First, to examine the genetic and environmental aetiologies of low extreme ADHD (SWAN) traits; second, to test the hypothesis that extreme deviations in either direction on ADHD (SWAN) traits represent maladaptive outcomes. The SWAN allows differentiation of two ADHD subdimensions: inattentiveness and hyperactivity‐impulsivity. Given evidence for partial genetic separation, as well as differential correlates of these subdimensions (Greven et al., 2011, 2011), results were also explored separately for each (see Appendix S1).

## Method

### Sample

Participants were part of the Twins Early Development Study (TEDS), a U.K. population‐representative sample of twins born in England and Wales between 1994 and 1996 (Haworth, Davis, & Plomin, 2013). Families were excluded following severe pre‐ or perinatal complications, a severe medical condition in twins (e.g. a chromosomal disorder, brain damage, global developmental delay, autism, blindness, death of either twin), and if sex or zygosity were uncertain. This study included 2,143 twins (mean age 16.32 years; *SD* 0.68; range 14.91–18.76) from 1083 twin pairs: 415 monozygotic (MZ; male: 137, female: 278), 349 dizygotic (DZ) same‐sex (male: 132, female: 217), and 319 DZ opposite‐sex pairs. Informed written consent was obtained from the parents and twins (Institute of Psychiatry ethics approval PNM09 10‐104). Questionnaires were pencil‐and‐paper based and returned via free‐post envelope, some were web‐based (highlighted below).

### Measures

#### ADHD traits

Twins provided self‐reports on the 18 DSM‐IV‐based items of the Strength and Weaknesses of ADHD Symptoms and Normal behaviour (SWAN) questionnaire (Hay et al., 2007). Each item is phrased to represent behaviours on a continuum (e.g. sustains attention) rather than a symptom (e.g. difficulty sustaining attention), rated on a 7‐point Likert scale from (1) ‘far below’, (2) ‘below’, (3) ‘slightly below’, (4) ‘average’, (5) ‘slightly above’, (6) ‘above’ to (7) ‘far above’. Scores were reversed so that higher scores indicated more symptomatic ADHD traits. The SWAN has adequate reliability and validity. Using the continuously distributed SWAN has been shown to increase power in genome‐wide association studies (GWAS) compared to skewed measures or binary clinical cut‐offs (Van der Sluis, Posthuma, Nivard, Verhage, & Dolan, 2013). In childhood, ADHD behaviours are usually assessed through parent or teacher‐ratings; in late adolescence and adulthood, more commonly through self‐ratings. Previous twin studies investigating the entire distribution of individual differences found evidence for heritability of 69–90% of the SWAN when using parent‐ratings (Arnett et al., 2011; Ebejer et al., 2015; Polderman et al., 2007), and 30–69% when using child self‐report (Ebejer et al., 2015), whereas shared environmental contributions were nonsignificant. These estimates are similar in size to established measures of ADHD. One study also found significant shared environmental influences on the SWAN in a subset of analyses (ranging from non‐significant to 66%) reducing the variance attributable to heritability (31–89%) (Hay et al., 2007).

In the present sample, the distribution approximated normality (skew = 0.09, kurtosis = 0.01; Figure B.S1 in Appendix S2). Cronbach's alpha internal consistency was high (0.93). To test that low and high SWAN scores were both reliable, a mean split was used showing that Cronbach's alpha internal consistency was similar for below (0.91) or above (0.93) average SWAN scores. Moreover, previous research has shown that low‐bound estimates of test‐retest reliability, estimated by correlating SWAN scores across time points for MZ twins, are good for the SWAN (0.72–0.90) (Arnett et al., 2011).

#### Behavioural outcomes

##### Behaviour problems

Child externalizing and internalizing problems were assessed using parent and self‐ratings on the conduct problems items of the SDQ (Goodman & Scott, 1999), self‐ratings on the emotional symptoms items of the SDQ (Goodman & Scott, 1999) and parent‐ratings on the Mood and Feelings Questionnaire (MFQ) (Angold et al., 1995).

##### Positive traits

Child prosocial behaviour was assessed through parent‐ and self‐report on the prosocial items of the SDQ (Goodman & Scott, 1999). Child life satisfaction and happiness were assessed using self‐ratings on a shortened 21‐item web‐based Multidimensional Students’ Life Satisfaction Scale (Huebner, 1994) and the Subjective Happiness Scale (Lyubomirsky & Lepper, 1999). Child perseverance and passion for long‐term goals was assessed using self‐ratings on the web‐based Short Grit Scale (Duckworth & Quinn, 2009). Further, parent‐ and child‐reports (Yes/No) were gathered on the questions (Vital, Ronald, Wallace, & Happé, 2009): Does he/she display (do you feel you have) a striking skill, compared to his/her (your) general ability in other areas? Does he/she display (do others tell you that you have) a special ability, superior even to most adults?

#### Cognitive performance outcomes

School grades in English and Mathematics were obtained from postal questionnaire and telephone interview (for details see (Krapohl et al., 2014)) assessing the highest grade achieved in the General Certificate of Secondary Education (GCSE), an academic qualification taken by students in England, Wales and Northern Ireland at the end of compulsory education, or in alternative equivalent qualifications if GCSEs were not taken. Child general cognitive ability was assessed using a mean composite of the Mill Hill Vocabulary Test (Raven, Raven, & Court, 1998) and Ravens’ Standard Progressive Matrices (Raven, Court, & Raven, 1996), gathered via validated web‐based testing (Haworth et al., 2013).

#### Home environmental outcomes

Parents’ use of negative discipline strategies (e.g. shouting, smacking or slapping) was assessed using a child‐rated 4‐item web‐based questionnaire (Viding, Fontaine, Oliver, & Plomin, 2009). The web‐based child‐rated Confusion, Hubbub and Order Scale (CHAOS) scale assessed disorganization in the home environment (Matheny, Wachs, Ludwig, & Phillips, 1995).

### Analyses

To examine the aetiologies of extreme ADHD traits (aim 1), DF extremes analyses were conducted on low‐ and high extreme 5%, 10%, 15% and 20% cut‐offs on the SWAN, using Mx (Boker et al., 2011). DF extremes analysis is based on differential regression of ADHD scores of co‐twins of ADHD probands to the population mean for ADHD, where ADHD probands are selected for having extreme low or high ADHD traits (DeFries & Fulker, 1988). Group heritability (h^2^g) addresses to what extent genetic factors explain why those at the extreme, as a group, differ from the rest of the population. Likewise group‐shared (c^2^g) and nonshared (e^2^g) environment can be estimated, which refer to environmental factors contributing to twin similarity and dissimilarity respectively. We also obtained A (heritability), C (shared environmental) and E (nonshared environmental) estimates for the entire distribution of individual differences in ADHD traits using full‐information maximum likelihood estimation on raw data in Mx, creating residual scores, corrected for sex and age by means of regression (McGue & Bouchard, 1984). Because of a lack of power to examine sex differences within the extreme scoring groups, the standard approach was taken to exclude opposite‐sex twins from analyses for aim 1.

To test the hypothesis that extreme deviations in either direction on ADHD traits represent maladaptive outcomes (aim 2), polynomial regression analyses were conducted including the linear and nonlinear (quadratic) terms of ADHD traits as predictors (centred scores), age and gender as covariates, and behavioural, cognitive and home environmental measures as outcomes. Linear relations between ADHD traits and outcomes would indicate low extreme ADHD traits represent more adaptive outcomes than high‐extreme ADHD traits. Curvilinear relations could suggest low and high‐extreme traits are both associated with maladaptive outcomes, depending on the shape of the curve. Further, mean outcome measure scores were plotted of individuals with the highest and lowest 10% scores on the ADHD trait distribution, and children scoring in the average 10%. Outcome measures were standardized into z‐scores. The skewed SDQ and MFQ measures transformed using Van der Waerden transformation. Corrections were applied for the nonindependence of data due to including twin pairs (‘cluster’ command in STATA (Williams, 2000)) and multiple testing (False Discovery Rate; α at 0.05 (Benjamini & Hochberg, 1995)).

## Results

### DF extremes analysis of low and high‐extreme ADHD traits

**Figure 1:**
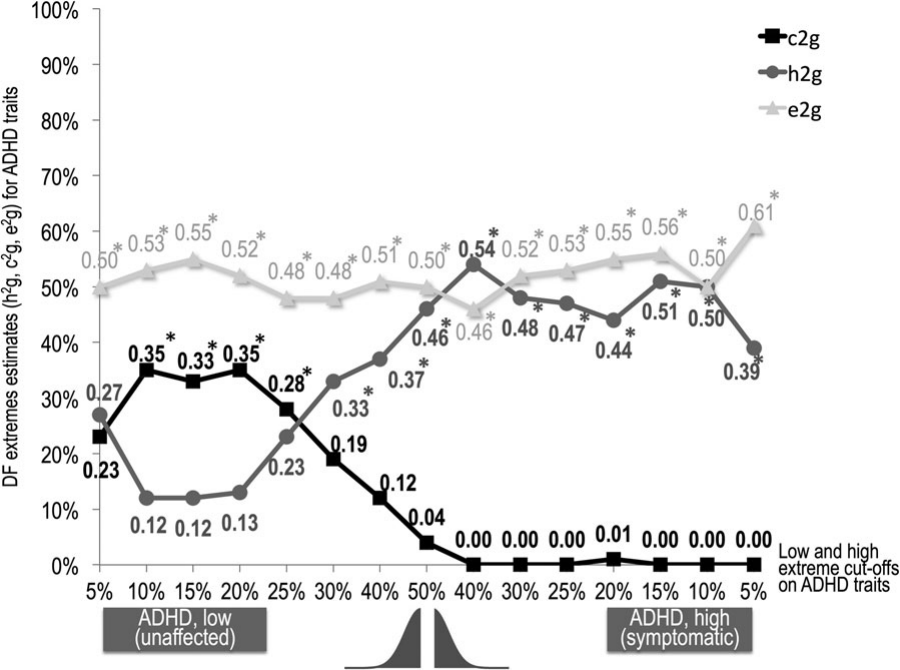
DeFries‐Fulker extremes estimates for attention deficit hyperactivity disorder traits at low and high‐extreme cut‐offs. *Estimate is significant (based on 95% confidence intervals; see Table 1)

DeFries–Fulker extremes analyses showed that, across 5%, 10%, 15% and 20% cut‐offs, h^2^g was significant for high extreme (h^2^g = 39–51%), but not for low extreme ADHD traits. In contrast, c^2^g was nonsignificant for high extreme, but significant for low extreme ADHD traits (c^2^g = 23–35%; exception: c^2^g was nonsignificant at the low 5% cut‐off, due to the smaller sample). However, estimates of h^2^g and c^2^g at low and high extremes did not differ significantly as 95% confidence intervals overlapped (Table 1). e^2^g, was significant for both high (e^2^g = 49–61%) and low (e^2^g = 50–55%) extremes (Table 1). Results were similar after correction for age and gender (Table B.S1, Appendix S2). Post hoc, we ran DF extremes analyses for less extreme cut‐offs. At 30%, 40% and 50% low extreme cut‐offs, c^2^g of low extreme ADHD traits was no longer significant, whereas h^2^g reached significance (Figure 1).

**Table 1:** Results of DeFries‐Fulker (DF) extremes analyses for ADHD traits using low and high 5%, 10%, 15% and 20% extreme cut‐offs

	Cut‐off, %	*N* probands (individuals)		Proband mean		Co‐twin mean		Twin group correlation		DF extreme estimate		
		MZ	DZ	MZ	DZ	MZ	DZ	MZ	DZ	h^2^g (95% CI)	c^2^g (95% CI)	e^2^g (95% CI)
ADHD, low (unaffected)	5	41	38	1.51	1.53	2.35	2.64	0.51	0.35	0.27 (0.00;0.65)	0.23 (0.00;0.52)	0.50 (0.35;0.65)
	10	82	74	1.76	1.77	2.53	2.66	0.48	0.39	0.12 (0.00;0.46)	0.35 (0.07;0.52)	0.53 (0.41;0.64)
	15	130	110	1.94	1.93	2.64	2.75	0.46	0.37	0.12 (0.00;0.44)	0.33 (0.07;0.50)	0.55 (0.44;0.65)
	20	165	133	2.04	2.01	2.64	2.75	0.49	0.40	0.13 (0.00;0.44)	0.35 (0.10;0.32)	0.52 (0.42;0.62)
ADHD, high (symptomatic)	5	33	41	4.90	4.87	3.95	3.46	0.43	0.14	0.39 (0.19;0.51)	0.00 (0.00;0.14)	0.61 (0.49;0.73)
	10	73	82	4.61	4.61	3.96	3.52	0.53	0.20	0.50 (0.30;0.61)	0.00 (0.00;0.15)	0.50 (0.39,0.61)
	15	93	102	4.52	4.53	3.90	3.55	0.51	0.24	0.51 (0.28;0.61)	0.00 (0.00;0.17)	0.49 (0.39;0.60)
	20	142	155	4.36	4.37	3.73	3.53	0.44	0.26	0.44 (0.13;0.55)	0.01 (0.00;0.25)	0.55 (0.45;0.66)

ADHD = ADHD total score on the SWAN measure. h^2^g = group heritability; c^2^g = group‐shared environment; e^2^g = group nonshared environment. Twin group correlation = the extent to which the mean standardized quantitative trait score of co‐twins is as low/high as the mean standardized score of probands selected for low/high ADHD traits. Doubling the difference in MZ and DZ group twin correlation gives a rough estimate h^2^g. 95% confidence intervals (CIs) that include zero indicate nonsignificance; nonoverlapping CIs indicate two estimates differ significantly.

Genetic (A = 46%) and nonshared environmental (E = 48%) estimates for the entire distribution of individual differences in ADHD traits were significant, whereas shared environmental influences were nonsignificant (C = 6%) (Table B.S2 in Appendix S2).

### Do low and high‐extreme ADHD traits represent maladaptive outcomes?

**Figure 2:**
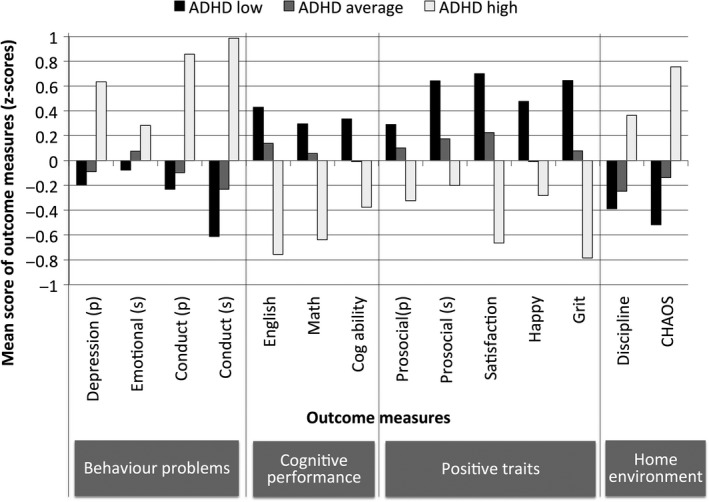
Mean behavioural, cognitive and home environmental outcome scores for groups scoring low, average and high on ADHD traits. p = parent‐rated. s = self‐rated. ADHD low = individuals falling into the low 10% (10th percentile) of the ADHD trait distribution on the SWAN measure, that is those with the lowest ADHD traits; ADHD average = individuals falling into the average 10% (45–55th percentile) of the distribution. ADHD high = individuals falling into the top 10% (90th percentile) of the ADHD trait distribution. Mean behavioural, cognitive and home environmental outcome scores were tabulated for low, average and high ADHD traits. Means calculated after randomly selecting one twin per pair to account for the nonindependence of data (results were similar for the co‐twins). Inspection of Figure 2 confirmed impressions from the regression analyses (see Table S2a,b in Supporting Information) in showing that those with the lowest ADHD scores had lower internalizing and externalizing behaviour scores, higher academic and cognitive performance, showed more positive traits and reported less harsh discipline and less chaotic homes compared to those at the high extreme. Individuals with average levels of ADHD traits tended to score intermediate to those at either extreme

**Figure 3:**
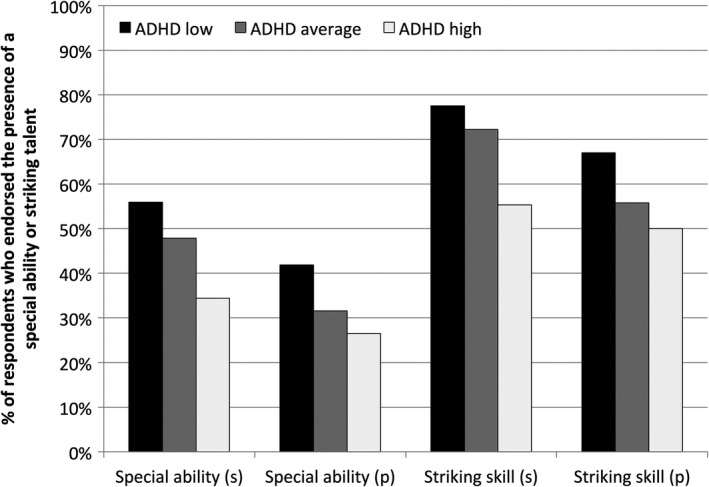
Percentage of respondents who reported a special ability of striking skill for groups scoring low, average and high on attention deficit hyperactivity disorder traits. Percentages obtained after randomly selecting one twin per pair to account for the nonindependence of data (results were similar for the co‐twins). See Figure 2 for an explanation of abbreviations

Polynomial regression analyses revealed that ADHD traits showed significant linear associations with all outcomes measures, whereas curvilinear associations were nonsignificant (the only exception was that five measures showed significant linear as well as curvilinear associations, as explained in Table B.S3 and Figure B.S2 in Appendix S2). Individuals at the low extreme of ADHD traits, relative to those at the high extreme, had lower internalizing and externalizing behaviour scores, higher general cognitive ability and school grades in English and Mathematics, showed more prosocial behaviour, greater life satisfaction, happiness and grit, were more likely to have a special ability or striking skill, and reported fewer negative parental discipline strategies and less confusion and disorganization at home. Individuals with average levels of ADHD traits tended to score intermediate to those at either extreme of the SWAN distribution (Figures 2 and 3). Individuals at low and high 10% extremes differed between 0.36 and 1.60 standard deviations (*SD*s) of a standard normal distribution (*M* = 0.00, *SD* = 1.00) on behaviour problems, between 0.71 and 1.19 *SD*s on cognitive performance, between 0.61 and 1.4 *SD*s on the continuous child positive traits, and 0.75 and 1.3 *SD*s on home environmental outcomes, indicating moderate to large effect sizes (Figure 2).

## Discussion

This study examined the genetic and environmental aetiologies of low extreme ADHD traits, and tested the hypothesis that low and high‐extreme ADHD trait scores may both be maladaptive. As a main novel finding, and in contrast to our hypothesis, low extreme ADHD traits were not significantly heritable, but were significantly influenced by group‐shared (23–35%) and nonshared environment (50–55%). This is a striking finding as shared environmental influences on high extreme and individual differences in ADHD traits are typically nonsignificant and close to zero (Burt, 2009; Willcutt, 2005). Consistent with the literature (Larsson et al., 2012; Willcutt, 2005), high‐extreme ADHD traits showed significant group heritability (39–51%) and group nonshared environmental (49–61%), but nonsignificant group‐shared environmental effects; estimates that resembled genetic and environmental contributions to individual differences in ADHD traits. This suggests that there are genetic links between the high distribution extreme and normal variation in ADHD traits.

We found no evidence to support an evolutionary trade‐off hypothesis (Nettle, 2006), that is that those scoring in the average range would show the most favourable outcomes. Instead, low extreme ADHD traits, compared to the high extreme or average levels of ADHD traits, were related to fewer behaviour problems, better cognitive performance, more positive traits, less chaotic homes and less negative parental discipline. Results are consistent with a recent study in a population sample of 378 children (6–13 years) which found lower SWAN scores to be linked to fewer parent‐rated internalizing and externalizing behaviour problems and better performance on neurocognitive tasks (Greven et al., 2015); and a study using the SWAN in a community sample (ages 6–18 years) which showed that individuals with the lowest possible SWAN score performed better on the stop signal task (assessing response inhibition, response latency and response variability) than those with the highest possible score (Crosbie et al., 2013).

What do these findings mean for a ‘positive genetics’ model of ADHD? First, above the low 30% cut‐off (Figure 1), group heritability of ADHD traits reached significance and group‐shared environment became nonsignificant, suggesting a turning point at which influences of genetic and shared environmental factors changes over the continuum. Genetic and environmental effects do not operate in isolation, but interact in the form of gene‐environment interaction and correlation (r_GE_). Hence, the effect of genes and environments on ADHD traits may be nonlinear across the continuum of ADHD traits. For example, in the presence of perinatal insults, influences of genetic factors on problem behaviour have been shown to decrease and those of shared and nonshared environmental factors to increase, suggestive of environmental moderation of heritability (Wichers et al., 2002). A potential mechanism explaining our findings is passive r_GE_, which arises because biological relatives provide environments as well as passing on genes and which inflates the shared environmental component (Plomin et al., 2013). Rather than reflecting a single environmental source, we speculate this may be a result of a combination of positive family environmental influences such as high SES, adaptive parenting styles and nutritional factors. Direct evidence for passive r_GE_ would arise if the correlation between such family environmental outcomes and low ADHD traits was greater in nonadoptive than adoptive families (Rijsdijk & Sham, 2002).

Second, our findings suggest that there may be additional, shared environmental, factors contributing to variation in low extreme ADHD traits that are not operating across the normal variation in ADHD traits or high‐extreme traits, consistent with the discontinuity hypothesis. This, in combination with the nonsignificant group heritability, suggests that extremely low ADHD traits may not be connected with the construct we know as ADHD at the high extreme or represent different aetiological mechanisms. This requires further investigation in larger samples, as estimates of group heritability and shared environment did not differ significantly between opposite ADHD trait extremes, hence the continuity hypothesis could not be rejected entirely. Of note, power to detect shared environment is lower in twin studies than that to detect heritability, adding further emphasis to the role of shared environmental influences on low extreme traits.

Third, finding nonsignificant group heritability could also be consistent with reduced reliability in measurement of the low extreme. However, similar internal consistency of below and above average SWAN scores (see Method) and the pretty level group nonshared environment estimates (which include measurement error) across the distribution suggest that there is no difference in measurement error at high and low ends, supporting the quality of the measurement at the low extreme.

Fourth, shared environment in ADHD deserves further attention. Typically influence of shared environment decreases with age, and tends to be minimal for ADHD, whereas heritability increases (Burt, 2009; Plomin et al., 2013). Participants were in their midadolescence, hence finding influences of shared environment on ADHD traits is striking. In contrast to nonshared environments and genetic effects, shared environmental influences represent a more readily identifiable and persistent source of variability in childhood and adolescence (Burt, 2014). If we can identify positive and protective environments influencing low ADHD traits, this could provide clues for relevant factors that could be targeted in interventions for those with clinically elevated ADHD scores or to prevent individuals from growing into deficit.

### Strengths and limitations

This is the first study to test the group heritability of low extreme ADHD traits and uses an established twin sample. Findings are based on adolescents’ self‐report and should be replicated in childhood, using parent‐ and teacher‐report, and in a larger sample. Using self‐report is also a strength as self‐reported ADHD traits are relatively underexplored in twin research (Merwood et al., 2013). Whether behaviours at the low extreme form a stable trait and represent resilience, that is, reduced vulnerability to environmental adversity (Rutter, 2012), was not addressed in this study. Hence, whether those at low extreme do better or relatively well even in the face of experiencing risk remains unclear. Future studies could test this using propensity score matching to test if children differing only in being low or high ADHD traits, but not on other risks (e.g. SES, parental diagnostic status), have different outcomes. Standard assumptions and limitations of the twin method applied to this study (Plomin et al., 2013).

### Conclusions and implications

Continuously distributed measures of ADHD provide value beyond improved psychometric properties and advantages of parametric testing, through allowing assessment of previously poorly measured behaviours at the low extreme. A focus on the low extreme might be relevant if we wish to nurture individual strengths, helping those with positive ADHD‐related behaviours to reach their full potential. This is different from the current, deficit‐based model of intervening only to assist those with high‐extreme ADHD traits or at risk of adverse psychosocial outcomes, but is congruent with a population‐level approach to improving youth wellbeing (Sanders et al., 2008).

**Key points**

- Mental disorders such as ADHD are assessed through binary diagnosis or skewed measures biased towards the high, symptomatic extreme. This violates the fundamental assumption that mental disorders are multifactorial and normally distributed, and has tempered research progress.
- Overcoming this methodological limitation, this is the first study to assess the aetiologies of low extreme ADHD traits in a population sample of twins.
- In contrast to the high extreme, extremely low ADHD traits represented better‐than‐average adaptive behaviours and cognition. These low traits were significantly influenced by shared and nonshared environmental, but not genetic factors.
- The study paves the way for new possibilities to study mechanisms underlying previously neglected positive behaviours.

## Supporting information


**Appendix S1.** Subdimensions of ADHD traits.
**Appendix S2.** ADHD traits (total score).
