# Supplementary material for: The opposite end of the attention deficit hyperactivity disorder continuum: genetic and environmental aetiologies of extremely low ADHD traits
Source: J Child Psychol Psychiatry. 2015 Oct 17;57(4):523–31. doi: 10.1111/jcpp.12475 (PMC4789118; doi:10.1111/jcpp.12475)
Supplement: Supplementary file 1 — Appendix S1. Subdimensions of ADHD traits. Appendix S2. ADHD traits (total score). [file JCPP-57-523-s001.docx]

**Supporting information for *The opposite end of the ADHD continuum: genetic and environmental aetiologies of extremely low ADHD traits by* Greven et al.**

**Table of contents**

Appendix A. subdimensions of ADHD traits (inattentiveness and hyperactivity-impulsivity) 2

Text box A.S1. Summary of results for *inattentiveness* and *hyperactivity-impulsivity* 2

Fig. A.S1. Mean behavioural, cognitive and home environmental outcome scores for groups scoring low, average and high on *inattentiveness*. 3

Fig. A.S2. Mean behavioural, cognitive and home environmental outcome scores for groups scoring low, average and high on *hyperactivity-impulsivity*. 4

Fig. A.S3. Percentage of respondents who reported a special ability of striking skill for groups scoring low, average and high on *inattentiveness*. 5

Table A.S1. Results of DF extremes analyses for *inattentiveness* using low and high 5%, 10%, 15% and 20% extreme cut-offs 7

Table A.S2. Results of DF extremes analyses for *hyperactivity-impulsivity* using low and high 5%, 10%, 15% and 20% extreme cut-offs 8

Table A.S3. Heritability (A), shared environment (C) and non non-shared environmental (E) influences on *inattentiveness* and *hyperactivity-impulsivity* across the entire range of individual differences 9

Table A.S4. Polynomial regressions of *inattentive*ness on outcome measures 10

Table A.S5. Polynomial regressions of *hyperactivity-impulsivity* on outcome measures 12

Appendix B. ADHD traits (total score) 14

Fig. B.S1. Distribution of *ADHD traits*, assessed using the SWAN scale. 14

Fig. B.S2. Scatterplot of linear and quadratic relations. 15

Table B.S1. Results of DF extremes analyses for *ADHD traits* using low and high 5%, 10%, 15% and 20% extreme cut-offs, after correction of ADHD traits for age and gender 16

Table B.S2. Heritability (A), shared environment (C) and non non-shared environmental (E) influences on *ADHD traits* across the entire range of individual differences 17

Table B.S3. Polynomial regressions of *ADHD traits* on outcome measures 18

Appendix A. Online supporting information for subdimensions of ADHD traits (inattentiveness and hyperactivity-impulsivity)

Text box A.S1. Summary of results for *inattentiveness* and *hyperactivity-impulsivity*

Cronbach’s alpha for inattentiveness (0.89) and hyperactivity-impulsivity (0.90) were good.

DF extremes analyses revealed that, overall, the pattern of significant h^2^g for the high extreme, significant c^2^g for the low extreme, and significant e^2^g for high and low extremes was replicated for both ADHD dimensions, with some exceptions (Tables A.S1-A.S2): Notably, c^2^g ranged from 23-41% for low hyperactivity-impulsivity, and from 16-27% for low inattentiveness; however only at the 15% cut-off was the estimate significant for inattentiveness. This suggests that the significant c^2^g for total ADHD traits may be more strongly driven by hyperactivity-impulsivity. Inattentiveness (A=48%, C=2%, E=45%) and hyperactivity-impulsivity (A=46%, C=3%, E=51%) showed significant heritability across the entire range of individual differences, whereas shared environmental contributions were non-significant (Table A.S3).

Polynomial regression analyses revealed that the two ADHD dimensions showed significant linear associations with all outcomes measures, similar to the total score of ADHD traits, only the strength of the linear association (see proportions of variance explained) was somewhat smaller for hyperactivity-impulsivity than inattentiveness (with the exception of conduct problems) (Tables A.S4-A.S5). Further, individuals with average levels of inattentiveness or hyperactivity-impulsivity traits also tended to score intermediate to those at either extreme (Fig. A.S1-A.S4).

Fig. A.S1. Mean behavioural, cognitive and home environmental outcome scores for groups scoring low, average and high on *inattentiveness*.

p=parent-rated. s=self-rated. INATT low= individuals falling into the low 10% (10th percentile) of the inattentive trait distribution, i.e. those with the lowest inattentiveness; INATT average= individuals falling into the average 10% (45-55th percentile) of the distribution. INATT high= individuals falling into the top 10% (90th percentile) of the inattentive trait distribution. Mean behavioural, cognitive and home environmental outcome scores were tabulated for low, average and high inattentiveness. Means calculated after randomly selecting one twin per pair to account for the non-independence of data (results were similar for the co-twins).


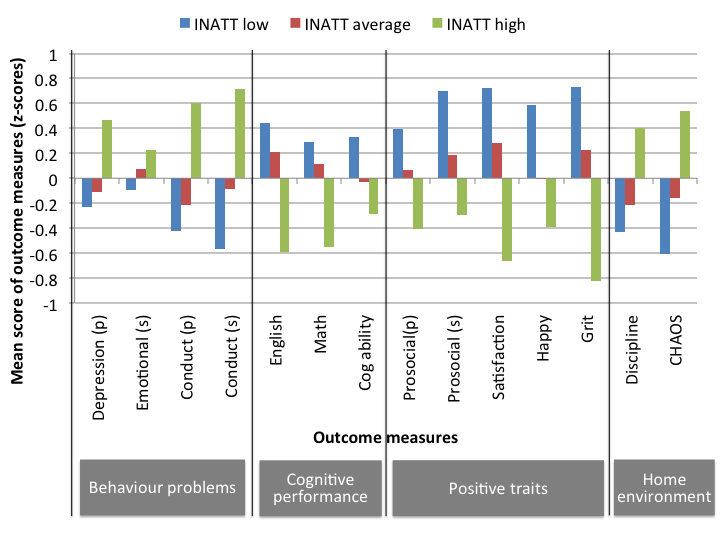


Fig. A.S2. Mean behavioural, cognitive and home environmental outcome scores for groups scoring low, average and high on *hyperactivity-impulsivity*.

p=parent-rated. s=self-rated. HYP-IMP low= individuals falling into the low 10% (10th percentile) of the hyperactive-impulsive trait distribution, i.e. those with the lowest hyperactivity-impulsivity; HYP-IMP average= individuals falling into the average 10% (45-55th percentile) of the distribution. HYP-IMP high= individuals falling into the top 10% (90th percentile) of the hyperactive-impulsive trait distribution. Mean behavioural, cognitive and home environmental outcome scores were tabulated for low, average and high hyperactivity-impulsivity. Means calculated after randomly selecting one twin per pair to account for the non-independence of data (results were similar for the co-twins).

**
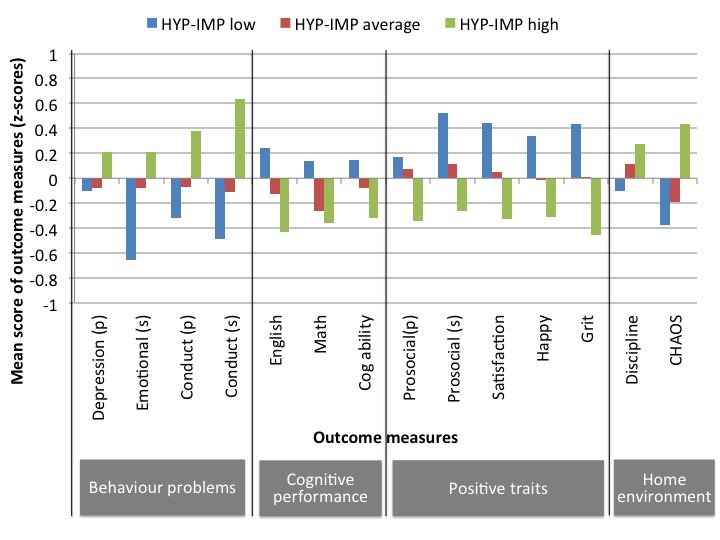
**

Fig. A.S3. Percentage of respondents who reported a special ability of striking skill for groups scoring low, average and high on *inattentiveness*.

Percentages obtained after randomly selecting one twin per pair to account for the non-independence of data (results were similar for the co-twins). See Fig A.S1 for an explanation of abbreviations.


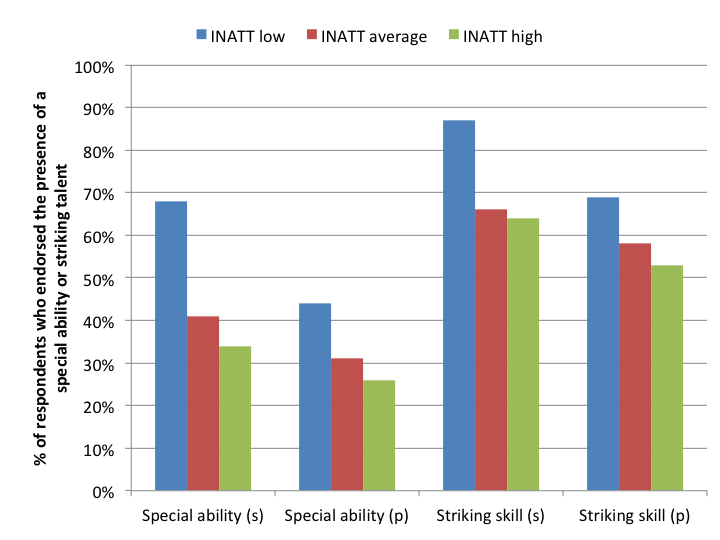


Fig. A.S4. Percentage of respondents who reported a special ability of striking skill for groups scoring low, average and high on hyperactivity-impulsivity.

Percentages obtained after randomly selecting one twin per pair to account for the non-independence of data (results were similar for the co-twins). See Fig A.S2 for an explanation of abbreviations. Contrary to the significant Odds Ratios (ORs) for striking skill in Table A.S5 (ORs below 1, suggesting that lower hyperactivity-impulsivity is linked to higher chance for having a striking skill), those in the ‘HYP-IMP low’ category had a slightly smaller percentage of respondents reporting a striking skill. A likely explanation is the weak association between hyperactivity-impulsivity and having a striking skill, in combination with the reduced power when looking within percentiles.


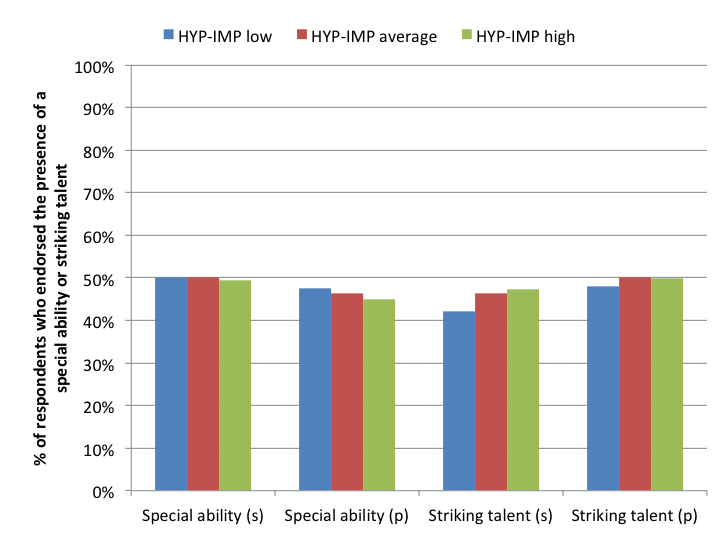


Table A.S1. Results of DF extremes analyses for *inattentiveness* using low and high 5%, 10%, 15% and 20% extreme cut-offs

|  | Cut-off | N probands (individuals) | | Proband mean | | Co-twin mean | | Twin group correlation | | DF extreme estimate | | |
| --- | --- | --- | --- | --- | --- | --- | --- | --- | --- | --- | --- | --- |
|  |  | MZ | DZ | MZ | DZ | MZ | DZ | MZ | DZ | h^2^g (95% CI) | c^2^g (95% CI) | e^2^g (95% CI) |
| INATT, low (unaffected) |  |  |  |  |  |  |  |  |  |  |  |  |
|  | 5% | 36 | 33 | 1.46 | 1.54 | 2.39 | 2.66 | 0.50 | 0.35 | 0.21 (0.00; 0.65) | 0.27 (0.00; 0.55) | 0.52 (0.34; 0.69) |
|  | 10% | 80 | 76 | 1.75 | 1.81 | 2.55 | 2.81 | 0.49 | 0.31 | 0.25 (0.00; 0.59) | 0.22 (0.00; 0.48) | 0.53 (0.40; 0.66) |
|  | 15% | 123 | 113 | 1.94 | 1.96 | 2.59 | 2.86 | 0.52 | 0.32 | 0.31 (0.00; 0.60) | 0.19 (0.07; 0.45) | 0.50 (0.39; 0.61) |
|  | 20% | 153 | 130 | 2.03 | 2.02 | 2.61 | 2.89 | 0.53 | 0.31 | 0.37 (0.05; 0.62) | 0.16 (0.00; 0.41) | 0.48 (0.37; 0.58) |
| INATT, high (symptomatic) |  |  |  |  |  |  |  |  |  |  |  |  |
|  | 5% | 32 | 39 | 5.04 | 4.02 | 3.89 | 3.57 | 0.35 | 0.16 | 0.34 (0.00; 0.48) | 0.00 (0.00; 0.14) | 0.66 (0.52; 0.80) |
|  | 10% | 60 | 73 | 4.78 | 4.77 | 3.94 | 3.62 | 0.44 | 0.22 | 0.44 (0.15; 0.56) | 0.00 (0.00; 0.15) | 0.56 (0.44, 0.68) |
|  | 15% | 75 | 92 | 4.69 | 4.68 | 3.89 | 3.57 | 0.43 | 0.20 | 0.42 (0.17; 0.54) | 0.00 (0.00; 0.19) | 0.57 (0.46; 0.69) |
|  | 20% | 122 | 147 | 4.49 | 4.49 | 3.78 | 3.56 | 0.41 | 0.23 | 0.42 (0.13; 0.53) | 0.00 (0.00; 0.22) | 0.58 (0.47; 0.69) |

Note. INATT = inattentiveness scale on the SWAN measure.

Table A.S2. Results of DF extremes analyses for *hyperactivity-impulsivity* using low and high 5%, 10%, 15% and 20% extreme cut-offs

|  | Cut-off | N probands (individuals) | | Proband mean | | Co-twin mean | | Twin group correlation | | DF extreme estimate | | |
| --- | --- | --- | --- | --- | --- | --- | --- | --- | --- | --- | --- | --- |
|  |  | MZ | DZ | MZ | DZ | MZ | DZ | MZ | DZ | h^2^g (95% CI) | c^2^g (95% CI) | e^2^g (95% CI) |
| HYP-IMP, low (unaffected) |  |  |  |  |  |  |  |  |  |  |  |  |
|  | 5% | 47 | 38 | 1.26 | 1.24 | 2.33 | 2.42 | 0.44 | 0.39 | 0.08 (0.00; 0.51) | 0.36 (0.01; 0.52) | 0.56 (0.42; 0.69) |
|  | 10% | 64 | 58 | 1.35 | 1.37 | 2.37 | 2.43 | 0.44 | 0.41 | 0.03 (0.00; 0.41) | 0.41 (0.10; 0.52) | 0.56 (0.43; 0.67) |
|  | 15% | 146 | 119 | 1.68 | 1.68 | 2.49 | 2.65 | 0.46 | 0.36 | 0.18 (0.00; 0.49) | 0.28 (0.02; 0.48) | 0.54 (0.44; 0.64) |
|  | 20% | 171 | 139 | 1.76 | 1.76 | 2.50 | 2.69 | 0.48 | 0.34 | 0.24 (0.00; 0.55) | 0.23 (0.00; 0.47) | 0.53 (0.42; 0.63) |
| HYP-IMP, high (symptomatic) |  |  |  |  |  |  |  |  |  |  |  |  |
|  | 5% | 42 | 46 | 5.11 | 5.10 | 4.25 | 3.47 | 0.55 | 0.15 | 0.50 (0.28;0.63) | 0.00 (0.00; 0.15) | 0.50 (0.37; 0.64) |
|  | 10% | 70 | 69 | 4.86 | 4.89 | 4.06 | 3.47 | 0.52 | 0.17 | 0.49 (0.28; 0.60) | 0.00 (0.00; 0.15) | 0.51 (0.40, 0.63) |
|  | 15% | 89 | 82 | 4.75 | 4.80 | 4.02 | 3.50 | 0.53 | 0.20 | 0.51 (0.31; 0.62) | 0.00 (0.00; 0.15) | 0.49 (0.38; 0.60) |
|  | 20% | 145 | 139 | 4.52 | 4.54 | 3.88 | 3.47 | 0.52 | 0.22 | 0.51 (0.30; 0.61) | 0.00 (0.00; 0.16) | 0.49 (0.39; 0.60) |

Note. HYP-IMP= hyperactivity-impulsivity scale on the SWAN measure.

Table A.S3. Heritability (A), shared environment (C) and non non-shared environmental (E) influences on *inattentiveness* and *hyperactivity-impulsivity* across the entire range of individual differences

|  | INATT | HYP-IMP |
| --- | --- | --- |
| rMZ | 0.48 (0.40; 0.55) | 0.48 (0.41; 0.55) |
| rDZ | 0.28 (0.18; 0.38) | 0.27 (0.16; 0.36) |
| A (CI) | 0.48 (0.26; 0.57) | 0.46 (0.23; 0.55) |
| C (CI) | 0.02 (0.00; 0.21) | 0.03 (0.00; 0.22) |
| E (CI) | 0.49 (0.49; 0.43) | 0.51 (0.45; 0.59) |

*Note.* INATT = inattentiveness scale, HYP-IMP= hyperactivity-impulsivity scale on the SWAN measure. rMZ = monozygotic twin correlation, rDZ= dizygotic twin correlation. 2*(rMZ – rDZ) gives a rough estimate of individual differences heritability. CI= 95% confidence interval.

Table A.S4. Polynomial regressions of *inattentive*ness on outcome measures

|  |  | **Behaviour problems** | | | | | | | |  | **Cognitive performance** | | | | | |  | **Home environmental outcomes** | | | |
| --- | --- | --- | --- | --- | --- | --- | --- | --- | --- | --- | --- | --- | --- | --- | --- | --- | --- | --- | --- | --- | --- |
|  |  | **Depression scores** (parent-rating) | | **Emotional symptoms** (self-rating) | | **Conduct problems** (parent-rating) | | **Conduct problems** (self-rating) | |  | **English**  (school grade) | | **Mathematics**  (school grade) | | **General cognitive ability**  (cognitive test) | |  | **Parental discipline**  (self-rating) | | **CHAOS**  (self-rating) | |
|  |  | B | R^2^/ ∆R^2^ | B | R^2^/ ∆R^2^ | B | R^2^/ ∆R^2^ | B | R^2^/ ∆R^2^ |  | B | R^2^/ ∆R^2^ | B | R^2^/ ∆R^2^ | B | R^2^/ ∆R^2^ |  | B | R^2^/ ∆R^2^ | B | R^2^/ ∆R^2^ |
| Step 0: |  |  | R^2^=0.0086 |  | R^2^=0.1105 |  | R^2^=0.0016 |  | R^2^=0.0050 |  |  | R^2^=0.0283 |  | R^2^=0.0042 |  | R^2^=0.0016 |  |  | R^2^=0.0006 |  | R^2^=0.0874 |
| Step 1: |  |  | ∆R^2^=0.0325 |  | ∆R^2^=0.0169 |  | ∆R^2^=0.0717 |  | ∆R^2^=0.1099 |  |  | ∆R^2^=0.0655 |  | ∆R^2^=0.0810 |  | ∆R^2^=0.0253 |  |  | ∆R^2^=0.0303 |  | ∆R^2^=0.0883 |
|  | INATT | 0.19*** |  | 0.16*** |  | 0.31*** |  | 0.37*** |  |  | -0.34*** |  | -0.32*** |  | -0.17*** |  |  | 0.19*** |  | 0.34*** |  |
| Step 2: |  |  | ∆R^2^=0.0056 |  | ∆R^2^=0.0014 |  | ∆R^2^=0.0811 |  | ∆R^2^=0.0039 |  |  | ∆R^2^=0.0041 |  | ∆R^2^=0.0042 |  | ∆R^2^=0.0000 |  |  | ∆R^2^=0.0014 |  | ∆R^2^=0.0000 |
|  | INATT | 0.19*** |  | 0.16*** |  | 0.30*** |  | 0.37*** |  |  | -0.34*** |  | -0.33*** |  | -0.17*** |  |  | 0.19*** |  | 0.34*** |  |
|  | INATT^2^ | 0.07** |  | 0.04 |  | 0.08*** |  | 0.06** |  |  | -0.06 |  | -0.06* |  | -0.00 |  |  | 0.04 |  | 0.01 |  |

**Table A.S4 (continued). Polynomial regressions of *inattentive*ness on outcome measures**

|  |  | **Positive traits** | | | | | | | | | |  | | |  |
| --- | --- | --- | --- | --- | --- | --- | --- | --- | --- | --- | --- | --- | --- | --- | --- |
|  |  | **Prosocial behaviour**  (parent rating) | | **Prosocial behaviour**  (self-rating) | | **Life satisfaction**  (self-rating) | | **Happiness**  (self-rating) | | **Grit**  (self-rating) | | **Special ability**  (self-rating) | **Special ability**  (parent-rating) | **Striking skill**  (self-rating) | **Striking skill**  (parent-rating) |
|  |  | B | R^2^/ ∆R^2^ | B | R^2^/ ∆R^2^ | B | R^2^/ ∆R^2^ | B | R^2^/ ∆R^2^ | B | R^2^/ ∆R^2^ | OR (CI)† | OR (CI)† | OR (CI)† | OR (CI)† |
| Step 0: |  |  | R^2^=0.0232 |  | R^2^=0.0810 |  | R^2^=0.0022 |  | R^2^=0.0006 |  | R^2^=0.0053 |  |  |  |  |
| Step 1: |  |  | ∆R^2^=0.0354 |  | ∆R^2^=0.1492 |  | ∆R^2^=0.1264 |  | ∆R^2^=0.0605 |  | ∆R^2^=0.2099 |  |  |  |  |
|  | INATT | -0.23*** |  | -0.28*** |  | -0.39*** |  | -0.31*** |  | -0.54*** |  | 0.67***  (0.59-0.75) | 0.72***  (0.64-0.81) | 0.61***  (0.54-0.69) | 0.74** *  (0.66-0.83) |
| Step 2: |  |  | ∆R^2^=0.0004 |  | ∆R^2^=0.1514 |  | ∆R^2^=0.0004 |  | ∆R^2^=0.0003 |  | ∆R^2^=0.0000 |  |  |  |  |
|  | INATT | -0.23*** |  | -0.28*** |  | -0.40*** |  | -0.31*** |  | -0.54*** |  | 0.66***  (0.59-0.74) | 0.71***  (0.63-0.81) | 0.62***  (0.55-0.70) | 0.74***  (0.66-0.83) |
|  | INATT^2^ | -0.02 |  | 0.04 |  | -0.02 |  | 0.02 |  | -0.00 |  | 0.99  (0.90-1.09) | 0.93  (0.84-1.04) | 0.97  (0.88-1.07) | 1.03  (0.94-1.12) |

*Note.* INATT = inattentiveness scale on the SWAN measure. INATT^2^=quadratic term of INATT. B=unstandardised regression coefficient. OR=odds ratio (1df), CI=95% confidence interval. Step 0 includes: age, gender. Step 1 includes: age, gender, INATT. Step 2 includes: age, gender, INATT, INATT^2^. R^2^=% of variance explained by age and gender. ∆R^2^=incremental % of variance explained by INATT (beyond age, gender; step 1), or by INATT^2^ (beyond age, gender, INATT; step 2). Results corrected for multiple testing using the False Discovery Rate (α at 0.05). †based on logistic regression. ***p<0.001, **p<0.01, *p<0.05.

Table A.S5. Polynomial regressions of *hyperactivity-impulsivity* on outcome measures

|  |  | **Behaviour problems** | | | | | | | |  | **Cognitive performance** | | | | | |  | **Home environmental outcomes** | | | |
| --- | --- | --- | --- | --- | --- | --- | --- | --- | --- | --- | --- | --- | --- | --- | --- | --- | --- | --- | --- | --- | --- |
|  |  | **Depression scores** (parent-rating) | | **Emotional symptoms** (self-rating) | | **Conduct problems** (parent-rating) | | **Conduct problems** (self-rating) | |  | **English**  (school grade) | | **Mathematics**  (school grade) | | **General cognitive ability**  (cognitive test) | |  | **Parental discipline**  (self-rating) | | **CHAOS**  (self-rating) | |
|  |  | B | R^2^/ ∆R^2^ | B | R^2^/ ∆R^2^ | B | R^2^/ ∆R^2^ | B | R^2^/ ∆R^2^ |  | B | R^2^/ ∆R^2^ | B | R^2^/ ∆R^2^ | B | R^2^/ ∆R^2^ |  | B | R^2^/ ∆R^2^ | B | R^2^/ ∆R^2^ |
| Step 0: |  |  | R^2^=0.0086 |  | R^2^=0.1105 |  | R^2^=0.0016 |  | R^2^=0.0050 |  |  | R^2^=0.0283 |  | R^2^=0.0042 |  | R^2^=0.0016 |  |  | R^2^=0.0053 |  | R^2^=0.0009 |
| Step 1: |  |  | ∆R^2^=0.0160 |  | ∆R^2^=0.0033 |  | ∆R^2^=0.0462 |  | ∆R^2^=0.1219 |  |  | ∆R^2^=0.0126 |  | ∆R^2^=0.0234 |  | ∆R^2^=0.0159 |  |  | ∆R^2^=0.0609 |  | ∆R^2^=0.0503 |
|  | HYP-IMP | 0.10*** |  | 0.06** |  | 0.21*** |  | 0.34*** |  |  | -0.17*** |  | -0.13*** |  | -0.11*** |  |  | 0.11*** |  | 0.22*** |  |
| Step 2: |  |  | ∆R^2^=0.0049 |  | ∆R^2^=0.0002 |  | ∆R^2^=0.0132 |  | ∆R^2^=0.0105 |  |  | ∆R^2^=0.0101 |  | ∆R^2^=0.0128 |  | ∆R^2^=0.0051 |  |  | ∆R^2^=0.0001 |  | ∆R^2^=0.0032 |
|  | HYP-IMP | 0.10*** |  | 0.06** |  | 0.22*** |  | 0.34*** |  |  | -0.18*** |  | -0.14*** |  | -0.11*** |  |  | 0.11*** |  | 0.23*** |  |
|  | HYP-IMP^2^ | 0.05** |  | 0.01 |  | 0.09*** |  | 0.08*** |  |  | -0.09* |  | -0.10*** |  | -0.06* |  |  | 0.00 |  | 0.04 |  |

**Table A.S5 (continued). Polynomial regressions of *hyperactivity-impulsivity* on outcome measures**

|  |  | **Positive traits** | | | | | | | | | |  | | |  |
| --- | --- | --- | --- | --- | --- | --- | --- | --- | --- | --- | --- | --- | --- | --- | --- |
|  |  | **Prosocial behaviour**  (parent rating) | | **Prosocial behaviour**  (self-rating) | | **Life satisfaction**  (self-rating) | | **Happiness**  (self-rating) | | **Grit**  (self-rating) | | **Special ability**  (self-rating) | **Special ability**  (parent-rating) | **Striking skill**  (self-rating) | **Striking skill**  (parent-rating) |
|  |  | B | R^2^/ ∆R^2^ | B | R^2^/ ∆R^2^ | B | R^2^/ ∆R^2^ | B | R^2^/ ∆R^2^ | B | R^2^/ ∆R^2^ | OR (CI)† | OR (CI)† | OR (CI)† | OR (CI)† |
| Step 0: |  |  | R^2^=0.0232 |  | R^2^=0.0810 |  | R^2^=0.0022 |  | R^2^=0.0006 |  | R^2^=0.0609 |  |  |  |  |
| Step 1: |  |  | ∆R^2^=0.0075 |  | ∆R^2^=0.0521 |  | ∆R^2^=0.0647 |  | ∆R^2^=0.0367 |  | ∆R^2^=0.0662 |  |  |  |  |
|  | HYP-IMP | -0.11*** |  | -0.21*** |  | -0.24*** |  | -0.21*** |  | -0.25*** |  | 0.84*** (0.76-0.92) | 0.87**  (0.78-0.96) | 0.82**  (0.74-0.90) | 0.86**  (0.78-0.95) |
| Step 2: |  |  | ∆R^2^=0.0010 |  | ∆R^2^=0.0010 |  | ∆R^2^=0.0000 |  | ∆R^2^=0.0024 |  | ∆R^2^=0.0001 |  |  |  |  |
|  | HYP-IMP | -0.11*** |  | -0.21*** |  | -0.24*** |  | -0.21*** |  | -0.25*** |  | 0.84***  (0.77-0.92) | 0.86**  (0.78-0.96) | 0.82***  (0748-0.91) | 0.86**  (0.78-0.95) |
|  | HYP-IMP^2^ | -0.02 |  | 0.02 |  | 0.00 |  | 0.04 |  | -0.00 |  | 1.07  (1.00-1.15) | 0.98  (0.90-1.06) | 1.07  (0.99-1.16) | 1.05  (0.98-1.13) |

*Note.* HYP-IMP = hyperactivity-impulsivity scale on the SWAN measure. HYP-IMP^2^=quadratic term of HYP-IMP. B=unstandardised regression coefficient. OR=odds ratio (1df), CI=95% confidence interval. Step 0 includes: age, gender. Step 1 includes: age, gender, HYP-IMP. Step 2 includes: age, gender, HYP-IMP, HYP-IMP^2^. R^2^=% of variance explained by age and gender. ∆R^2^=incremental % of variance explained by HYP-IMP (beyond age, gender; step 1), or by HYP-IMP^2^ (beyond age, gender, HYP-IMP; step 2). Results corrected for multiple testing using the False Discovery Rate (α at 0.05). †based on logistic regression. ***p<0.001, **p<0.01, *p<0.05.

Appendix B. Online supporting information for ADHD traits (total score)

Fig. B.S1. Distribution of *ADHD traits*, assessed using the SWAN scale.

Typically, interview and questionnaire measures of ADHD show skewed distributions that attenuate variability at the low, unaffected end of the distribution. In contrast, the SWAN resulted in an approximately normal distribution in the TEDS population-based sample and might therefore be regarded to assess ADHD traits on a continuum from low to high.

*High extreme, symptomatic*

*Low extreme, unaffected*

Fig. B.S2. Scatterplot of linear and quadratic relations.

The figure illustrates the J- rather than U-shape of the quadratic curve using child-ratings on conduct problems as an example. Scatterplots for the other outcome measures showing significant quadratic relations with ADHD traits (see Table B.S3) showed similar J-shaped curves.


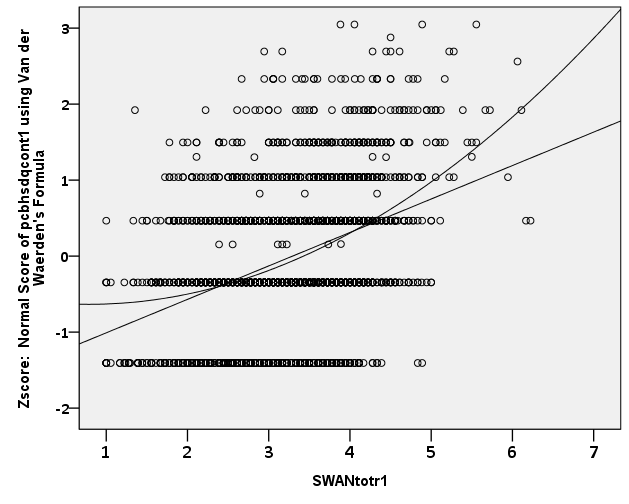


**SWAN total score**

**Conduct problems (self-ratings, z-score)**

**Total SWAN score**

Table B.S1. Results of DF extremes analyses for *ADHD traits* using low and high 5%, 10%, 15% and 20% extreme cut-offs, after correction of ADHD traits for age and gender

|  | Cut-off | N probands (individuals) | | Proband mean | | Co-twin mean | | Twin group correlation | | DF extreme estimate | | |
| --- | --- | --- | --- | --- | --- | --- | --- | --- | --- | --- | --- | --- |
|  |  | MZ | DZ | MZ | DZ | MZ | DZ | MZ | DZ | h^2^g (95% CI) | c^2^g (95% CI) | e^2^g (95% CI) |
| ADHD, low (unaffected) |  |  |  |  |  |  |  |  |  |  |  |  |
|  | 5% | 40 | 37 | -1.75 | -1.72 | -0.86 | -0.62 | 0.49 | 0.35 | 0.22 (0.00; 0.62) | 0.26 (0.00; 0.52) | 0.52 (0.37; 0.67) |
|  | 10% | 77 | 72 | -1.52 | -1.50 | -0.72 | -0.59 | 0.47 | 0.39 | 0.10 (0.00; 0.45) | 0.36 (0.07; 0.52) | 0.54 (0.42; 0.65) |
|  | 15% | 124 | 103 | -1.34 | -1.35 | -0.63 | -0.51 | 0.46 | 0.37 | 0.12 (0.00; 0.45) | 0.33 (0.06; 0.50) | 0.55 (0.44; 0.66) |
|  | 20% | 171 | 137 | -1.20 | -1.23 | -0.62 | -0.48 | 0.50 | 0.40 | 0.18 (0.00; 0.48) | 0.32 (0.07; 0.52) | 0.50 (0.40; 0.60) |
| ADHD, high (symptomatic) |  |  |  |  |  |  |  |  |  |  |  |  |
|  | 5% | 33 | 42 | 1.65 | 1.61 | 0.73 | 0.20 | 0.45 | 0.13 | 0.40 (0.22;0.52) | 0.00 (0.00; 0.13) | 0.60 (0.48; 0.71) |
|  | 10% | 67 | 80 | 1.38 | 1.37 | 0.68 | 0.26 | 0.50 | 0.20 | 0.47 (0.26; 0.60) | 0.00 (0.00; 0.15) | 0.53 (0.41, 0.64) |
|  | 15% | 105 | 115 | 1.22 | 1.23 | 0.59 | 0.29 | 0.49 | 0.25 | 0.49 (0.23; 0.60) | 0.00 (0.00; 0.20) | 0.51 (0.40; 0.61) |
|  | 20% | 143 | 158 | 1.10 | 1.10 | 0.45 | 0.26 | 0.42 | 0.25 | 0.43 (0.12; 0.54) | 0.01 (0.00; 0.25) | 0.56 (0.46; 0.67) |

*Note.* ADHD=ADHD total score on the SWAN measure. See the note to Table 1 in the main manuscript for an explanation of table contents.

Table B.S2. Heritability (A), shared environment (C) and non non-shared environmental (E) influences on *ADHD traits* across the entire range of individual differences

|  | ADHD |
| --- | --- |
| rMZ | 0.49 (0.42; 0.57) |
| rDZ | 0.30 (0.20; 0.39) |
| A (CI) | 0.46 (0.24; 0.58) |
| C (CI) | 0.06 (0.00; 0.24) |
| E (CI) | 0.48 (0.42; 0.56) |

*Note.* ADHD=ADHD total score on the SWAN measure. rMZ = monozygotic twin correlation, rDZ= dizygotic twin correlation. 2*(rMZ – rDZ) gives a rough estimate of individual differences heritability. CI= 95% confidence interval.

Table B.S3. Polynomial regressions of *ADHD traits* on outcome measures

|  |  | **Behaviour problems** | | | | | | | |  | **Cognitive performance** | | | | | |  | **Home environmental outcomes** | | | |
| --- | --- | --- | --- | --- | --- | --- | --- | --- | --- | --- | --- | --- | --- | --- | --- | --- | --- | --- | --- | --- | --- |
|  |  | **Depression scores** (parent-rating) | | **Emotional symptoms** (self-rating) | | **Conduct problems** (parent-rating) | | **Conduct problems** (self-rating) | |  | **English**  (school grade) | | **Mathematics**  (school grade) | | **General cognitive ability**  (cognitive test) | |  | **Parental discipline**  (self-rating) | | **CHAOS**  (self-rating) | |
|  |  | B | R^2^/ ∆R^2^ | B | R^2^/ ∆R^2^ | B | R^2^/ ∆R^2^ | B | R^2^/ ∆R^2^ |  | B | R^2^/ ∆R^2^ | B | R^2^/ ∆R^2^ | B | R^2^/ ∆R^2^ |  | B | R^2^/ ∆R^2^ | B | R^2^/ ∆R^2^ |
| Step 0: |  |  | R^2^=0.0086 |  | R^2^=0.1105 |  | R^2^=0.0016 |  | R^2^=0.0050 |  |  | R^2^=0.0283 |  | R^2^=0.0042 |  | R^2^=0.0016 |  |  | R^2^=0.0006 |  | R^2^=0.0009 |
| Step 1: |  |  | ∆R^2^=0.0269 |  | ∆R^2^=0.1210 |  | ∆R^2^=0.0105 |  | ∆R^2^=0.1498 |  |  | ∆R^2^=0.0466 |  | ∆R^2^=0.0542 |  | ∆R^2^=0.0238 |  |  | ∆R^2^=0.0258 |  | ∆R^2^=0.0824 |
|  | ADHD | 0.17*** |  | 0.13*** |  | 0.32*** |  | 0.44*** |  |  | -0.31*** |  | -0.27*** |  | -0.17*** |  |  | 0.18*** |  | 0.34*** |  |
| Step 2: |  |  | ∆R^2^=0.0068 |  | ∆R^2^=0.0002 |  | ∆R^2^=0.0868 |  | ∆R^2^=0.0082 |  |  | ∆R^2^=0.0133 |  | ∆R^2^=0.0155 |  | ∆R^2^=0.0035 |  |  | ∆R^2^=0.0002 |  | ∆R^2^=0.0012 |
|  | ADHD | 0.18*** |  | 0.13*** |  | 0.32*** |  | 0.45*** |  |  | -0.33*** |  | -0.29*** |  | -0.18*** |  |  | 0.18*** |  | 0.35*** |  |
|  | ADHD^2^ | 0.03** |  | 0.02 |  | 0.12*** |  | 0.09*** |  |  | -0.13** |  | -0.14*** |  | -0.06 |  |  | 0.02 |  | 0.04 |  |

**Table B.S3 (continued). Polynomial regressions of *ADHD traits* on outcome measures**

|  |  | **Positive traits** | | | | | | | | | |  | | |  |
| --- | --- | --- | --- | --- | --- | --- | --- | --- | --- | --- | --- | --- | --- | --- | --- |
|  |  | **Prosocial behaviour**  (parent rating) | | **Prosocial behaviour**  (self-rating) | | **Life satisfaction**  (self-rating) | | **Happiness**  (self-rating) | | **Grit**  (self-rating) | | **Special ability**  (self-rating) | **Special ability**  (parent-rating) | **Striking skill**  (self-rating) | **Striking skill**  (parent-rating) |
|  |  | B | R^2^/ ∆R^2^ | B | R^2^/ ∆R^2^ | B | R^2^/ ∆R^2^ | B | R^2^/ ∆R^2^ | B | R^2^/ ∆R^2^ | OR (CI)† | OR (CI)† | OR (CI)† | OR (CI)† |
| Step 0: |  |  | R^2^=0.0232 |  | R^2^=0.0810 |  | R^2^=0.0022 |  | R^2^=0.0006 |  | R^2^=0.0053 |  |  |  |  |
| Step 1: |  |  | ∆R^2^=0.0235 |  | ∆R^2^=0.0719 |  | ∆R^2^=0.1134 |  | ∆R^2^=0.0585 |  | ∆R^2^=0.1483 |  |  |  |  |
|  | ADHD | -0.20*** |  | -0.30*** |  | -0.39*** |  | -0.32*** |  | -0.47*** |  | 0.71***  (0.63-0.80) | 0.76***  (0.67-0.86) | 0.67***  (0.59-0.76) | 0.76***  (0.68-0.86) |
| Step 2: |  |  | ∆R^2^=0.0008 |  | ∆R^2^=0.0016 |  | ∆R^2^=0.0003 |  | ∆R^2^=0.0014 |  | ∆R^2^=0.0003 |  |  |  |  |
|  | ADHD | -0.20*** |  | -0.30*** |  | -0.39*** |  | -0.31*** |  | -0.47*** |  | 0.71***  (0.63-0.80) | 0.74***  (0.65-0.85) | 0.67***  (0.58-0.75) | 0.77***  (0.68-0.86) |
|  | ADHD^2^ | -0.03 |  | 0.04 |  | -0.02 |  | 0.05 |  | 0.02 |  | 1.01  (0.91-1.12) | 0.92  (0.82-1.03) | 1.03  (0.92-1.14) | 1.07  (0.97-1.18) |

*Note.* ADHD=ADHD total scores on the SWAN measure. ADHD^2^=quadratic term of ADHD. B=unstandardised regression coefficient. OR=odds ratio (1df), CI=95% confidence interval. Step 0 includes: age, gender. Step 1 includes: age, gender, ADHD. Step 2 includes: age, gender, ADHD, ADHD^2^. R^2^=% of variance explained by age and gender. ∆R^2^=incremental % of variance explained by ADHD (beyond age, gender; step 1), or by ADHD^2^ (beyond age, gender, ADHD; step 2). ADHD traits showed significant linear associations with all outcomes (behaviour problems, cognitive performance, home environmental outcomes, positive traits), whereas curvilinear associations were non-significant. Linear relations indicate low extreme ADHD traits represent more adaptive outcomes than high extreme ADHD traits. The only exception was that five outcome measures (parent-rated depression, parent- and self-rated conduct problems and school grades in English and mathematics) showed significant linear as well as curvilinear relations with ADHD traits. U-shaped curvilinear relations would suggest low and high extreme traits may both be associated with maladaptive outcomes. However, these curvilinear relations were J- rather than U-shaped. J-shaped curvilinear relations suggest the low extreme is associated with more adaptive outcomes than the high extreme, but not with the lowest scores on depression or conduct problems, or the highest school grades. Inspection of these J-shaped associations revealed that moving down from the high to the low extreme, a point was reached at which individuals improved at a slower rate on depression scores, conduct problems and school grades (see Fig. B.S1). J-shaped relations may have been influenced by restriction of variance at the low extreme of these outcomes measures. Results corrected for multiple testing using the False Discovery Rate (α at 0.05). †based on logistic regression. ***p<0.001, **p<0.01, *p<0.05.
